# The direct and indirect effects of the COVID-19 pandemic on the mental health of confined youth

**DOI:** 10.1186/s40352-024-00267-8

**Published:** 2024-04-09

**Authors:** Lin Liu

**Affiliations:** Department of Sociology and Criminology & Law, University of Florida, Gainesville, FL USA

**Keywords:** COVID-19, Incarcerated youth, Mental health

## Abstract

The COVID-19 pandemic posed an unprecedented threat to the mental health of youth due to its attendant, drastic changes in everyday life brought about by restrictions such as social distancing and the cancelation of in-person classes. Although numerous articles have discussed the impact of the pandemic on youths’ mental health, most of them have been opinion pieces. This study used state-wide empirical data to quantify the direct and indirect effect of the pandemic on the mental health of confined youth, a vulnerable social group that is rarely represented in school survey data. Group comparisons of youth who entered juvenile justice facilities during pandemic and non-pandemic times were also conducted. Findings revealed that youth who entered residential facilities during the pandemic due to criminal offenses had higher rates of prior mental health problems and victimization. With major confounders controlled, multivariate regression results showed that the impact of the pandemic on confined youths’ mental health is indirect: it conditioned the effect of underage drinking on the youths’ mental health. Youth who were admitted into facilities during the pandemic were more likely to experience mental health problems than their peers who entered facilities during non-pandemic times. Implications for policymaking are discussed.

## Introduction

In March 2020, the World Health Organization (WHO) declared a COVID-19 pandemic (WHO, 2020). By 31 March 2021, there were more than 127 million confirmed infections worldwide, resulting in more than 2.5 million deaths (WHO, 2020). Protocols such as stay-at-home orders, social distancing, and closures of entire economic and social sectors were implemented throughout the United States, which drastically changed people’s everyday lives (CDC, 2021). A large body of quantitative studies revealed that the shutdown and social distancing protocols were associated with higher levels of stress and mental health issues among youth in the general population (Jones et al., 2022; Rosen et al., 2021; Samji et al., 2022). However, limited research was devoted to a particularly vulnerable segment of this population: youth incarcerated in juvenile justice facilities. According to the latest data from the Juvenile Residential Facility Census, the number of adolescents in juvenile justice facilities reached 25,014 in 2020 (Office of Juvenile Justice & Delinquency Prevention, 2020). Each day in 2020, an average of 69 adolescents were admitted into a juvenile justice residential facility. This incarcerated population faced unique challenges during the pandemic. Their fear of being infected by COVID-19 likely resulted in significant stress and anxiety (Wallace et al., 2020). Infection prevention and control measures—restricting access to other youth, canceling visits, limiting the time that they could spend outside, etc.—also likely took a toll on their mental health (Robinson et al., 2020).

Numerous articles have discussed the challenges of confined youth in the United States during the COVID-19 pandemic generally (Barnert, 2020; Reid et al., 2022). However, most were opinion pieces aimed at raising awareness of the potential needs of confined youth, highlighting the difference between a facility environment and a community, and calling for measures to protect the confined youth from infection (e.g., Barnert, 2020; Buchanan et al., 2020). Some studies also focused on providing workable guidelines for prevention protocols (e.g., Hewson et al., 2020; Thompson et al., 2022). However, no studies used large-scale data to measure the effect of this pandemic on mental health with confounders controlled.

To fill this glaring literature gap, I used state-wide juvenile justice data that covered both the pre-COVID pandemic period (hereafter, non-pandemic period) and the COVID pandemic period (hereafter, pandemic period) and examined three possible effects of the pandemic. First, I compared the profiles of youth admitted into residential facilities during the pandemic and non-pandemic periods to identify specific vulnerabilities of the pandemic youth cohort. Second, I compared non-pandemic and pandemic cases in multivariate regression models to parcel out the effect of the pandemic youths’ mental health when other confounders are controlled for. Third, I assessed whether the pandemic indirectly affected the mental health of confined youth by conditioning the impact of a traditional risk factor (such as drug use or mental health history), incorporating interaction terms in multivariate regression models.

### The impact of the COVID-19 pandemic on at-risk youth

Studies have shown that vulnerability to stressful environments such as the pandemic is not evenly spread among all youth (Debowska et al., 2022; Silliman et al., 2020). Youth with mental health struggles are particularly vulnerable to stressful situations and at higher risk of using non-adaptive coping skills such as escapist or venting behaviors. Adolescents with mental health disorders exhibited a higher tendency to resort to escapist and aggressive acts as coping skills (Dangelmaier et al., 2006; Fonseca-Pedrero et al., 2010; Jalbrzikowski et al., 2012; Liu et al., 2022. In their longitudinal study of adolescents in the general population, Lin and colleagues showed that youth whose mental health improved also improved their use of adaptive coping styles such as relaxation and physical recreation, whereas those who suffered persistent mental disorders had a tendency to use non-adaptive copings styles such as worrying and self-blaming in stressful situations (Lin et al., 2011).

The pandemic unquestionably made a drastic change in youths’ everyday lives (WHO, 2020). School shutdowns, social distancing, and the uncertainty over when things would return to normal were particularly challenging for them (Schwartz et al., 2021). For those with poor skills for coping with stress, the everyday life changes during the pandemic might have increased their likelihood of engaging in deviant behaviors. Studies of suicide rates during the pandemic suggested an association between suicide and several pandemic-related experiences such as social distancing and the fear of contracting the virus (Ammerman et al., 2021). There was also a rise in aggressive behavior during the business lockdown and stay-at-home orders (Barlett et al., 2021; Killgore et al., 2021). For example, Condry et al. (2020) surveyed 104 parents with children who had been aggressive toward their parents and 47 practitioners who had worked with families experiencing child/adolescent-to-parent violence. They found a 70% increase in violent episodes reported by parents and a 69% increase in referrals for these behaviors.

It is possible that youth struggling with mental health problems reacted to the pandemic-related challenges with non-adaptive coping behaviors such as violence, aggression, drug use, and other delinquent behaviors. If this was the case, the rate of youth with mental health struggles who engaged in delinquency and entered the juvenile justice system likely increased during the pandemic. The environment in juvenile justice facilities during the pandemic could also be stress-inducing (Buchanan et al., 2020). Across the country, juvenile justice agencies issued emergency orders that suspended visitation at juvenile residential commitment programs (Buchanan et al., 2020). Youth and their families communicated primarily by phone and video calls, but digital visits might not have yielded the same benefit for youth as in-person visits would have (Davidson et al., 2023). Also, education staff, counselors, and officers who work in juvenile justice facilities were screened every day prior to entering the facility; some of them might have been denied entry if they displayed flu-like symptoms (Barnert, 2020). Staff were not permitted to return to work until they had been cleared by a medical professional (Barnert, 2020). This process could have affected the activities scheduled for the youth, presenting them with the challenge of coping with last-minute cancelations and changes. This suggests that youth who stayed in juvenile justice facilities during the pandemic may have been at a higher risk of mental health struggles compared to their peers who stayed in facilities during non-pandemic periods.

Empirical tests on the effect of the pandemic on justice-involved youths’ mental health are still in their infancy. Using growth curve modeling, researchers have found that psychological distress and antisocial behavior increased among a sample of 557 youth in Florida who were under community supervision after the outbreak of COVID-19 (Reid et al., 2022). However, a study of prisoners in 31 United Kingdom prisons revealed that recorded incidents of self-harm decreased by one-third during the pandemic compared to the pre-pandemic rate (Hewson et al., 2020). The divergent results from these studies might be due to sample differences. Reid et al.’s (2022) small sample of youth under community supervision was considerably different from Hewson et al.’s (2020) sample of persons incarcerated in secured facilities. The pandemic may have affected youth under community supervision differently than those in a facility. No published study has quantified the effect of the pandemic on the mental health of confined youth in the United States. Another difference between the two studies is the scope of the confounders they controlled for. Reid et al.’s (2022) study focused on the change over time in mental health indicators among youth during the pandemic; confounders such as family support, past mental health history, and drug use history were not controlled. Hewson et al.’s (2020) study compared the total number of self-harming incidents before and after the pandemic; they employed descriptive rather than multivariate modeling techniques to quantify the effect of the pandemic.

### The current study

To date, no studies have used large-scale, state-wide data to compare confined youth who entered the juvenile justice facilities during the pandemic with those who entered in non-pandemic times. It is also not known if the perceived family support among youth who stayed in facilities during the pandemic is linked to their mental health. Lastly, the effect of the pandemic on justice-involved youths’ mental health has not been statistically distinguished from the effect of other factors such as adverse childhood experiences, mental health history, and substance use history.

This study leveraged four years of state-wide juvenile justice data from Florida, a large state with a racially diverse justice-involved youth population. The focus was on three potential effects of the pandemic. First, I examined whether youth with preexisting mental health struggles were more likely to demonstrate maladaptive behavior, in response to the sudden change in everyday life caused by the pandemic, that resulted in them being placed in juvenile justice residential facilities. Second, I assessed whether youth who had stayed in facilities during the pandemic had a higher risk of mental health struggles compared with their peers who had been confined in non-pandemic time. Third, I estimated the indirect effect of the pandemic on mental health during incarceration by testing whether another risk factor (substance use, mental health issues, and insufficient parental support) had an amplified, detrimental effect during the pandemic.

## Methods

### Data

The sample consisted of youth who entered a juvenile justice residential facility in Florida and received a Residential Assessment for Youth (RAY) (Florida Department of Juvenile Justice [FDJJ], 2021) between May 2019 and May 2022, which included both pandemic and non-pandemic times. FDJJ began using the RAY assessment tool in May 2019, so earlier data was not available. March 11, 2020 was used as the starting date of the pandemic because COVID-19 was officially designated a pandemic by the World Health Organization on this day (Daniel, 2023). March 25, 2022 was selected as the end date to include youth admitted during the pandemic but before mask mandates and other COVID-19 restrictions on businesses had been lifted that day (Markowitz & Rough, 2023). During this three-year period, 3,286 youth were admitted into residential facilities. The RAY assessment included multiple risks and needs factors such as criminal and victimization histories, drug and alcohol use, mental health, and family support. The assessment was administered by a bachelor-degreed, residential program case manager within 30 days after a youth was admitted into a facility. Case managers had been required to complete a standardized 2-day motivational interview training and a 3-day assessment and case planning training. Among the four race/ethnic categories in the sample—White, Black, Hispanic, and other—only 0.24% of the youth (*n* = 8) fell into the “other” category, making it unrealistic to compare the ‘other’ category with the White, Black and Hispanic categories. The cases coded “other” were excluded from the current study, resulting in a final sample of 3,278 youth.

### Measures

This study had two components. The first was a comparison of youth placed in juvenile justice facilities during the pandemic (between March 11, 2020 and March 25, 2022) with those placed during non-pandemic times (between May 1, 2019 and March 10, 2020), based on descriptive analyses. The second component was a comparison of non-pandemic and pandemic cases using multivariate regression to parcel out the direct and indirect effects of COVID on the mental health of confined youth. Several factor domains were used to build profiles of the youth who engaged in offending: mental health issues, substance use problems, and adverse childhood experiences. Mental health issues included three binary variables: history of anger and irritability (1 = yes, 0 = no), history of depression (1 = yes, 0 = no), and history of suicidal ideation (1 = yes, 0 = no). Substance misuse included two binary variables: drug use (1 = used drugs, 0 = did not use drugs) and alcohol consumption (1 = engaged in underage drinking, 0 = did not engage in underage drinking) before being admitted into the juvenile justice system. Adverse childhood experiences included nine binary variables: victim of neglect (1 = yes, 0 = no), sexually abused or raped by someone outside the family (1 = yes, 0 = no), sexually abused or raped by a family member (1 = yes, 0 = no), witness to violence at home (1 = yes, 0 = no), witness to a family member killed as a result of violence (1 = yes, 0 = no), witness to violence in a foster home (1 = yes, 0 = no), victim of physical abuse by a family member (1 = yes, 0 = no), victim of physical abused in a foster home (1 = yes, 0 = no), and victim of physical violence regardless of their relationship with the perpetrator (1 = yes, 0 = no).

The second component of the analysis involved regressing a mental health outcome variable on multiple predictors, including the pandemic. Mental health outcome, a binary variable, was measured by the RAY. A value of one was assigned if the youth was diagnosed with depression or anxiety at the time of assessment. The independent variables included age (at admission into a facility), race/ethnicity (1 = White, 2 = Black, 3 = Latino), gender (1 = male, 2 = female), drug use history (1 = yes, 0 = no), drinking history (1 = yes, 0 = no), depression history (1 = yes, 0 = no), anger/irritability history (1 = yes, 0 = no), and lack of parental support during the stay in residential facilities (1 = lack of support, 0 = presence of support). It should be noted that 148 (4.5%) of the youth reported that their parents showed little or no willingness to support them during their incarceration; another 18 (0.55%) said their parents were hostile, berating, and belittling. These two categories were merged due to the extremely small number in the second category. An additional predictor was the COVID-19 pandemic, a binary variable indicating whether the youth received the RAY risk/needs assessment during the pandemic (1 = yes, 0 = no).

### Analytic strategy

Descriptive statistics (i.e., frequency distributions) were calculated to describe the sample. A series of Chi-square tests were then conducted to measure the difference in mental health, substance use, and adverse childhood experience risk factors between the pandemic and non-pandemic cohorts. I then used logistic modeling to determine how the pandemic affected the mental health of youth in residential facilities. This involved regressing mental health indicators on the COVID-19 pandemic indicator and the selected confounders (e.g., mental health history, substance use history, and family support during youths’ stay in a facility). I also examined whether the confounders had an amplified detrimental effect on mental health that was contingent on the pandemic. This was achieved by a second logistic regression model, which estimated the interaction effects of the pandemic and traditional risk factors.

## Results

### Descriptive statistics

Table 1 reports the descriptive statistics for all variables used in this analysis. Of the 3,278 youth in the sample who had been admitted into a residential facility and assessed, 333 arrived during the pandemic (between March 11, 2020 and March 25, 2022), 2,945 before the pandemic (between May 1, 2019 and March 10, 2020). The largest proportion were non-Hispanic Black (60%), followed by non-Hispanic White (28%), and Hispanic (12%). Nearly 87% of the youth were male. The average age of the youth was 14 years; ages ranged from six to 18. Nearly 70% had a history of anger and irritability, which was higher than their rate of prior depression (44%). Victimization experiences were common: about 25% had witnessed violence at home, 19% had undergone physical abuse, and 15% had suffered from neglect. While residing in a facility, 69% experienced mental health issues (either anxiety or depression). About 5% reported a lack of parental support during their confinements.

**Table 1:** Descriptive Statistics (*n* = 3,278)

Variables	Mean or percentage	Std. Dev	Min	Max
Dependent variables				
Experiencing mental health issues during incarceration	68.55%			
Independent variables				
Admitted into a facility during the pandemic	10.16%			
History of Anger/Irritability	69.80%			
History of Depression/Anxiety	43.84%			
Underage drinking before admission	44.30%			
Substance misuse before admission	77.67%			
History of being a victim of neglect	15.25%			
Sexually abused by someone outside the family	6.13%			
Sexually abused by a family member	4.76%			
Witnessed a family member killed as a result of violence	2.17%			
Witnessed violence in a foster home	3.60%			
Witnessed violence at home	25.32%			
Physically abuse by a family member	4.27%			
Physically abused in a foster home	1.77%			
Physically abused in any relationship	18.91%			
Experienced suicidal ideation before admission	20.23%			
Lack of parental support during incarceration	5.06%			
Control variables				
Age	13.71	1.90	5.75	18.00
White	27.97%			
Black	60.04%			
Latinx	11.99%			
Female	13.06%			
Male	86.94%			

### Comparing the pandemic and non-pandemic cohorts

Several characteristics differentiated the youth who entered the juvenile justice system during the pandemic (see Table 2). Compared to those who had entered a juvenile justice facility in non-pandemic times, youth who entered during the pandemic were more likely to have a history of mental health issues. About 80% of the pandemic cohort had a history of anger and irritability, compared to 69% in the non-pandemic cohort (χ^2^ = 16.82, *p* < 0.001). The gap in the history of depression is smaller (19% v. 15%), but still reached statistical significance (χ^2^ = 23.97, *p* < 0.001). The difference in sexual abuse history between the two cohorts was not significant. Regarding exposure to violence, the pandemic cohort was more likely than the non-pandemic cohort to have witnessed a family member killed as a result of violence (2% v. 4%, χ^2^ = 3.61, *p* = 0.06) and violence at home (25% v. 31%, χ^2^ = 6.17, *p* < 0.01). The pandemic cohort also had a higher rate of experiencing neglect (15% v. 19%, χ^2^ = 3.85, *p* < 0.05). Experience with physical abuse also differentiated the two groups. Compared to the non-pandemic cohort, the pandemic cohort was more likely to have experienced physical abuse in a foster home (2% v. 4%, χ^2^ = 7.17, *p* < 0.01). The gap in the two groups’ experience of physical abuse by family members did not reach statistical significance. Lastly, a history of suicidal ideation did not differentiate the pandemic cohort—there was no statistically significant difference in rates between the two groups.

**Table 2:** Comparison of admission cases during non-pandemic and pandemic times

Measurements	Percentages		*p* value from chi-square tests
	Non-pandemic (*n* = 2,945)	Pandemic (*n* = 333)	
Experienced mental health problems during incarceration	68.05	72.97	*p* = .07
History of Anger/Irritability	68.69	79.58	*p* < .0001
History of Depression/Anxiety	42.41	56.46	*p* < .0001
Underage drinking before admission	44.04	46.55	*p* = .38
Substance misuse before admission	76.74	85.89	*P* < .0001
History of being a victim of neglect	14.84	18.92	*p* = .05
Sexually abused by someone outside the family	6.08	6.61	*p* = .70
Sexually abused by a family member	4.72	5.11	*p* = .75
Witnessed a family member killed as a result of violence	2.00	3.60	*p* = .06
Witnessed violence in a foster home	3.40	5.41	*p* = .06
Witnessed violence at home	24.69	30.93	*p* = .01
Physically abuse by a family member	4.24	4.50	*p* = .82
Physically abused in a foster home	1.56	3.60	*p* = .01
Physically abused in any relationship	18.57	21.92	*p* = .14
Experienced suicidal ideation before admission	19.90	23.12	*p* = .16
Lack of parental support during incarceration	5.23	3.60	*p* = .20

### The main effect of the pandemic on mental health issues of confined youth

Logistic regression Model 1 simultaneously estimated the effects of the pandemic and the confounders on the occurrence of mental health issues while the youth were incarcerated (see Table 3). The reference groups for race/ethnicity and gender were White and female, respectively. I started by estimating the pandemic variable. The results suggested the pandemic had a statistically significant effect. With confounders controlled, the odds of experiencing mental health issues were not statistically significantly different between the non-pandemic and pandemic cohorts (*OR* = 1.06, *p* = 0.56). Some confounders achieved significance. Youth who had a history of depression or anxiety were more likely to experience mental health issues while incarcerated than their peers with no history of depression or anxiety (*OR* = 1.95, *p* < 0.001 and *OR* = 1.74, *p* < 0.001, respectively). Interestingly, youth who had a history of drinking were less likely to have experienced mental health issues while staying in facilities (*OR* = 0.81, *p* < 0.01). Gender achieved marginal significance. Female youth were somewhat more likely to have experienced mental health issues in facilities than their male counterparts (*OR* = 1.25, *p* = 0.08). Other predictors such as lack of parental support, race/ethnicity, and age did not achieve statistical significance.

**Table 3:** Multivariate regression results predicting experiencing mental health issues during incarceration

**Parameters**	**Model 1**			**Model 2**		
	**OR**	**95%CI**	**SE**	**OR**	**95%CI**	**SE**
Admitted into a facility during the pandemic	1.08	0.83–1.41	0.14	0.97	0.21 4.47	0.78
Substance misuse before admission	1.06	0.87–1.29	0.10	1.11	0.91 - 1.37	0.10
Underage drinking before admission	0.81**	0.68–0.95	0.09	0.76**	0.64 - 0.91	0.09
History of Anger/Irritability	1.74***	1.47–2.07	0.09	1.69***	1.42 - 2.02	0.09
History of Depression/Anxiety	1.95***	1.63–2.32	0.09	2.03***	1.69 - 2.45	0.09
Lack of parental support during incarceration	1.22	0.87–1.72	0.17	1.19	0.83 - 1.69	0.18
Pandemic*Substance misuse history				0.53	0.24 - 1.19	0.41
Pandemic*Drinking history				1.73*	1.00 - 3.02	0.28
Pandemic*History of Anger/Irritability				1.41	0.75 - 2.66	0.32
Pandemic*History of Depression/Anxiety				0.67	0.38 - 1.18	0.29
Pandemic*Lack of parental support during incarceration				1.43	0.38 - 5.33	0.67
Age	0.99	0.95–1.03	0.02	0.99	0.95 - 1.03	0.02
Black	0.91	0.76–1.09	0.09	0.91	0.75 - 1.09	0.09
Latino	0.88	0.68–1.15	0.13	0.88	0.67 - 1.15	0.14
Female	1.25†	0.97–1.61	0.13	1.25†	0.97 - 1.62	0.13

The reference category of race/ethnicity is White. The reference category for gender is male

*OR* Odds ratio, *CI* Confidence interval, *SE* Standardized coefficients

^†^*p* < .1; *p < .05; **p < .01; ***p < .001

### The interaction effect of pandemic and traditional risk factors

Model 2 of Table 3 addressed whether the traditional risk factors of mental health exerted different effects if the youth entered a facility during the pandemic. Among the four interaction terms, we found that only pandemic*drinking history had exerted a statistically significant impact on the outcome (*OR* = 1.73, *p* < 0.001). The results suggest that although drinking seems to be associated with a lower risk of mental health issues while incarcerated, this alleviating effect declined during the pandemic. Among the non-pandemic cohort, drinking history was significantly associated with lower odds of having mental health issues while incarcerated (*OR* = 0.76, 95% confidence interval ranging from 0.64 to 0.91). However, I found no significant difference in the risk of mental health issues between youth with and without a drinking history during the pandemic (*OR* = 1.31, 95% confidence interval ranging from 0.78 to 2.24). Other interaction terms did not achieve statistical significance in the model.

## Discussion

The COVID-19 pandemic presented challenges for all groups of youth, especially those who are in correctional facilities. It prompted researchers, policymakers, and practitioners to think about how to best protect and support incarcerated youth should there be another pandemic. However, due to the fact that most research articles on this topic were opinion pieces aimed at raising awareness of the vulnerability of children to shutdown and social distancing protocols, we are facing a dearth of test results and research findings to quantify the impact of the pandemic and make evidence-driven policies. This study responded directly to this knowledge gap and used large-scale data to quantify the COVID-19 pandemic’s direct and indirect impact on incarcerated youth. The analyses showed that youth who were troubled by mental health issues, trauma, and abuse were less likely to properly adjust to the shutdown and social distancing protocols of the pandemic and more likely to react to these challenges by delinquency. Furthermore, the COVID-19 pandemic hampered the rehabilitation of youth in juvenile justice facilities.

First, the data revealed several characteristics that differentiated the group who entered the juvenile justice system during the pandemic from their peers who entered during non-pandemic times. The pandemic cohort was significantly more likely to have had a history of anxiety and depression, exposure to severe violence, and physical abuse. Several factors might help us understand why more youth with mental health and abuse issues entered the juvenile justice system during the pandemic. Their histories might have marginalized these youth from their school peers who hadn’t suffered from these types of issues (Hakulinen et al., 2020). When schools closed and switched to online instruction, these marginalized youth may not have had the necessary level of social interactions, since they no longer shared a physical space with their peers (Schwartz et al., 2021). With the online format, teens might have only kept contact with those they regarded as friends. Youth in the marginalized group, less likely to be deemed friends by others, would likely have suffered exacerbated isolation and loneliness (Hamilton et al., 2020).

Another potential factor to consider is the anxiety, perplexity, and paranoia caused by the overwhelming information related to COVID-19 during the pandemic. Much of the information circulating in social media and even some mainstream news outlets was misleading or false (Jurkowitz et al., 2020). This might have been particularly challenging for youth who already had mental health struggles as they attempted to cope with the confusion, uncertainty, and constantly evolving nature of new information and guidance about COVID-19. They might have found it more challenging to efficiently organize and appraise the information and to develop a reasonable plan for reducing their risk of exposure and infection. The anxiety, perplexity, and paranoia generated by overwhelming uncertainty may have increased the likelihood of deviant behaviors such as drug use or aggression and of ending up in the juvenile justice system.

Financial stress might also help explain why youth with a history of family abuse and violence were more likely to enter the juvenile justice system during the pandemic. The economic toll caused by the shutdown may have been more pronounced for youth in an abusive family. With the abuse and violence going on in the family or foster home, youth might have sought part-time jobs to supplement their cost of living (Liu & Miller, 2020). However, the mandated closure of businesses during the pandemic inevitably caused layoffs, especially among youth who were employed part-time and worked in service industries such as restaurants. With limited hope of being financially provided for by their abusive and violent caregivers/parents, youth might have resorted to crime to obtain income. Due to data limitations, it was beyond the scope of this study to examine the additional paths that may link abuse and mental health history to a higher risk of entering the juvenile justice system during the pandemic. Future studies should employ in-depth interviews with youth to ascertain how the life situations of disadvantaged youth might help us understand their criminal behavior during the pandemic.

The other major finding was the indirect effect of the COVID-19 pandemic on the mental health of confined youth. When multiple confounders were taken into account (such as past drug use and mental health problems), the pandemic was not found to have exerted a direct and significant effect on mental health during incarceration. However, it appears to have affected confined youth indirectly by conditioning the effect of traditional risk factors. Among youth who entered a facility in non-pandemic times, drinking behavior prior to admission was associated with a lower risk of having mental health issues while incarcerated. But during the pandemic, this alleviating effect of drinking disappeared. It is possible that the motive for drinking in non-pandemic times differed from that during pandemic times. In non-pandemic times, adolescents might drink for social reasons or to enhance their enjoyment (Kuntsche et al., 2005). For example, in a Canadian study, most students said they drank to enjoy the taste (24.9%), to celebrate (21.3%), or to be sociable (16.9%); only 2.1% drank to forget worries or feel less shy (Kairouz et al., 2002). If youth drinking has little to do with mental health and is more motivated by social reasons, youth who drink might have many friends (Jerez & Coviello, 1998). They might be less likely to experience mental struggles in residential facilities because they have the skills to make friends, build relationships, and avoid isolation and loneliness. However, during the pandemic, mandatory school shutdowns drastically changed the everyday lives of youth. Most social events and parties were canceled, so the motive for drinking might have become self-medication—to cope with stress and other negative emotions. Some studies found that alcohol consumption sharply increased during the pandemic due to the stress caused by the drastic changes in everyday life (Grossman et al., 2020; Nesoff et al., 2021). Thus, the youth who were found drinking underage before being incarcerated during the pandemic might also be more vulnerable to stress. They are distinct from the youth who drank in non-pandemic times for celebration and other social reasons and who had good skills for making friends and building relationships. This might explain why prior drinking among youth who were incarcerated in non-pandemic times predicted lower odds of mental struggles, while this effect was absent among the youth admitted during the pandemic.

While this study extended our understanding of the pandemic’s impact on a hard-to-reach social group, justice-involved youth, several limitations should be noted. First, the sample consisted of youth who had been placed in residential facilities after a conviction. The findings should not be generalized to delinquent youth who engaged in low-level delinquency and received diversion, community supervision, or other less severe sanctions. Second, due to data limitations, it was beyond the scope of this study to examine the pandemic’s effect on every type of mental disorder experienced by confined youth. The dataset only contained a binary indicator that measured whether a youth was experiencing depression or anxiety in a facility during the assessment. This measure is not granular, as there are more than a dozen types of mental health disorders including bipolar, schizophrenia, explosive emotional, and obsessive–compulsive disorders. Future studies should collect more specific data to examine whether the pandemic exerts the same impact on people with different types of mental disorders. The third limitation is that the data did not contain detailed operational indicators about the facilities such as which activities were canceled during the pandemic, the percentages of staff on sick leave, or the frequency the youth had phone/video calls with their parents. These environmental factors likely played a role in the mental health of the confined youth (Handwerk et al., 1998; Liu et al., 2024; Underwood et al., 2006). Future studies should collect data on these factors and assess their joint effects with individual risk factors.

The findings of the study identified several strategies policymakers may undertake should there be another pandemic. First, during a pandemic, alleviating the anxiety, uncertainty, and fear about a highly contagious disease confined youth experience should be a priority. The past COVID-19 pandemic presented enormous challenges for youth in secure facilities. Due to the distance away from school, community, and family, confined youth might find few avenues to collect information about the nature of the disease to evaluate their vulnerabilities to the disease. Timely and sufficient communications about the findings of a disease during a pandemic should occur between facility staff/officers and youth residents. Real-time updates about the facility’s response to the pandemic and measures taken should also be communicated to the youth. Such efforts may reduce the anxieties, fear, and uncertainty youth face. Second, a potential by-product of the pandemic is the social isolation that results from the suspension of in-person visits, meetings, and gatherings. For confined youth, a social group disproportionately suffering mental health problems and trauma history, social isolation can take a huge toll on their current mental health. During a pandemic, confined youth should have easy access to phone calls and mail services to maintain sufficient contact with their loved ones. During a time when in-person visitations are suspended, policymakers may consider utilizing technological advances and new virtual conference devices to provide high-quality virtual visitation time between confined adolescents and their family members.

## Data Availability

The quantitative data used in this study are received from the Florida Department of Juvenile Justice (FDJJ), which contains de-identified case records of the youth in the FDJJ system. Per FDJJ policy, case-level juvenile justice data are not to be publicly shared. However, there is a set of procedures that researchers can utilize to request data from FDJJ, which is on the FDJJ official website.
